# A review of recent treatments for adults living with attention-deficit/hyperactivity disorder

**DOI:** 10.4102/sajpsychiatry.v29i0.2152

**Published:** 2023-12-05

**Authors:** Candice Wakelin, Michele Willemse, Erica Munnik

**Affiliations:** 1 Department of Psychology, Faculty of Community and Health Sciences, University of the Western Cape, Cape Town, South Africa

**Keywords:** adult ADHD, intervention approaches, systematic review, treatment, SFS appraisal, evidence based interventions, RCT’s

## Abstract

**Background:**

Attention-deficit/hyperactivity disorder (ADHD) is a neuro-developmental disorder prevalent among children and adults. Adults living with ADHD can experience significant distress affecting their daily functioning on emotional, physical, interpersonal, familial and financial levels. Intervention programmes may be a way to mitigate these challenges.

**Aim:**

This review identified good evidence-based intervention studies for adults with ADHD and described the usefulness of these interventions.

**Method:**

Following Preferred Reporting Items for Systematic Reviews and Meta-Analyses (PRISMA) guidelines, articles were searched from 2009 to 2019 across four medical- and psychological-focused electronic databases using EBSCOhost. All articles selected for the review’s thematic meta-synthesis were appraised by attaining a threshold score of at least 61%, using the Smith-Franciscus-Swartbooi appraisal tool. Two autonomous reviewers engaged in the review process. The study adhered to all ethical principles pertaining to systematic review practice.

**Results:**

Forty studies were identified for summation, including pharmacological, non-pharmacological and neuro-stimulation approaches. Most interventions used a multimodal approach. Results indicated the most effective stimulant and non-stimulant as methylphenidate and atomoxetine, respectively. Effective non-pharmacological approaches to treatment were identified as cognitive-behavioural treatment, mindfulness-based approaches, psycho-education and dialectical-focused therapies. Bright light treatment and neurofeedback were reported as the most efficacious neuro-stimulatory methods.

**Conclusion:**

Pharmacological and non-pharmacological approaches, as well as neuro-stimulation or a blend of these approaches were acknowledged as the most effective recent modalities in the treatment of adult ADHD.

**Contribution:**

This review reported on the most current approaches to treat adult ADHD. This will facilitate a better understanding and informed decisions with regard to dealing with adult ADHD.

## Introduction

Attention-deficit/hyperactivity disorder (ADHD) is a disorder that appears in the Diagnostic and *Statistical Manual of Mental Disorders – Fifth Edition* (DSM-5) in the Chapter Neuro-developmental disorders.^1^ Studies show global prevalence rates between 2% and 5% for adults diagnosed with ADHD.^1,2^ Variability in occurrence still exists, in part because of symptom overlapping within psychiatric comorbidities, for example, mood and anxiety disorders, intermittent explosive disorders, substance use disorders and antisocial and other personality disorders.^1,3,4^ In addition, research shows that ADHD is globally under-emphasised and remains untreated in adults.^5,6^

Adult ADHD presents with the main symptoms of inattention, hyperactivity and impulsivity.^1^ Adults presenting with inattention have challenges in the domains of organisation, managing time effectively, ability to remain focused and usually struggles with the postponement of tasks. Hyperactivity in adults usually manifests as a proneness to fidgeting, excessive talking, inner hurry, overdoing and constant moving around. Impulsivity may be seen as an inability to think before acting, thus adults tend to make hasty decisions, interrupt others, experience difficulty waiting their turn during a conversation or answers questions before asked to do so.^1^ Impulsivity also impedes adults in foreseeing ‘what might happen’ or to anticipate what the immediate consequences of their actions are. For a diagnosis of ADHD, impulsivity, hyperactivity and inattention need to significantly disrupt daily activities in more than one context such as home and work. The disruption of life because of the symptoms is usually seen as unwanted by adults living with ADHD.^1^

As a result of the symptoms and disruptions, the lives of adults with ADHD are greatly affected on multiple levels. On an individual level, adults with ADHD tend to struggle with a range of associated psychological and executive problems, including a lack of self-image, challenges to regulate emotions effectively, increased irritability, difficulties with decision-making and discrepancies in recall and inhibition.^3^ These functional deficits contribute to individuals with ADHD experiencing high levels of daily stress, depression and even substance use disorders.^6^ On an interpersonal and familial level, adults may experience increased disharmony at home, family conflict and pessimism.^1,4^ Moreover, these individuals’ challenges with adequate attention to tasks often impacts family, friends and colleagues around them. Therefore, they might be described as indolent or noncompliant in the home and work contexts, thereby having an adverse impact on their relationships.^1,3^

The previously stated difficulties in family context often lead relatives to show an increased use of healthcare services, as research reports they experience psychological distress themselves, reflecting amplified levels of distress and high anxieties in daily living.^7,8^ In the work environment, ADHD in adults may result in low occupational performance, an increase in absenteeism from work and a greater likelihood of redundancy.^3,9^ As a result of the loss in competence and the increased nonattendance, this disorder has and continues to have a considerable economic effect.^10,11^ In addition, adults with ADHD use healthcare services more regularly than individuals without associated symptoms, thus producing increased treatment-related expenses.^12^

It is reported that studies focusing on the identification and treatment of adult ADHD have received sustained attention in recent years,^13,14^ as historically, the effect of ADHD on children and their relatives has been the focus of continuous local and international studies.^10,15^ Furthermore, there is a need for more focused interventions specifically aimed at assisting adults with ADHD to alleviate the challenges caused by this diagnosis on an individual level. Challenges also exist in the broader family and work context than merit attention.^13,14^

Generally, the term intervention is defined in literature as a ‘deliberate immersion in a process or system intended to impact events or consequences’,^13^ while a strategy is generally explained as ‘a plan of action designed to realise a longer term or general aim’.^13^ Interventions generally focus on long-term symptom reduction and an intervention approach usually entails the general approach mental health professionals take to improve an individual’s symptoms or assist the individual with optimal functioning.^13^ Intervention strategies’ purposes are generally to support the prevention of relapse, to treat comorbidity and to offer support to families. In this study, we decided to use intervention approaches as the overall framework to include programmes, strategies and intervention treatment modalities.

International studies conducted on interventions to help with adult ADHD mostly support a combination of pharmacological and non-pharmacological intervention approaches.^16,17,18^ Pharmacological treatments for ADHD include using either psychostimulants or non-stimulants. These depend on individual variability. Categories of psychostimulants include methylphenidate (MPH) and amphetamines, while non-stimulants include atomoxcitine (ATX), alpha-2-adrenoceptor agonists, tricyclic antidepressants (TCAs), bupropion, modafinil and venlafaxine.^11,16,17^

Although pharmacological treatments are generally considered as the preferred treatment for ADHD, not all individuals are keen to use medication. Possible reasons for this include a fear of stigma, worry over addiction, high prices of medication and many reported side effects. Individuals usually opt for alternative treatments such as psychosocial intervention approaches. According to research, psychosocial approaches are typically used to help to reduce ADHD symptoms in adults and mainly use cognitive-behavioural therapy (CBT), mindfulness-focused training (MBT), dialectical behaviour therapy (DBT), psychoeducation and coaching.^2,11,12^ Despite this information, evidence remains limited, and effectiveness is proven mainly for the more structured, skill-based CBT approaches used in combination with medication.^11^ Cognitive-behavioural therapy approaches generally assist to help individuals to take extra measures to anticipate and create external resources to back up existing memory losses. It also aims to decrease impulsivity, assist to regulate anger and improve management of time and organisational skills. They also aim to improve social and communication skills on an interpersonal level. Additionally, mindfulness exercises have been found to assist with improved emotional regulation and attention and are usually used as a complement for CBT or pharmacological treatments.^11,12,18^

Research with a focus on adult ADHD is still less prevalent than research performed on ADHD in children.^15,19,20^ Numerous authors identified the gap in the research pertaining to treatment approaches of ADHD in the adult population.^11,12,18,20^ This systematic review consolidated literature from 2009 to 2019 and focused on evidence-based intervention approaches for adults living with ADHD. The research questions investigated were:

- What are the methodological quality of the studies used in the review?
- Which intervention approaches are currently used in the treatment of adult ADHD?
- What are the outcomes of these approaches?

## Methods

### Design

The study used a systematic review as methodological design and considered peer-reviewed, full-text articles available in the public domain, with a focus on adult ADHD. The South African Bill of Rights^21^ describes adults as individuals aged 18 and older, and thus the sample population comprised male and females in this age group. The studies were quantitative studies drawing on observational designs (case-control and cross-sectional) and experimental designs (randomised control, as well as pre- and post-tests).

### Search procedure

The review was guided by the Preferred Reporting Items for Systematic Reviews and Meta-Analyses (PRISMA) reporting system.^22^ The PRISMA system recommends four review levels: (1) identification, (2) screening, (3) eligibility (quality appraisal) and (4) summation. Articles that focused on intervention strategies, models or programmes for adults with ADHD, published between 2009 and 2019, were open access, peer reviewed and written in English and drew on observational and experimental designs fulfilling the inclusion criteria of the review, were identified using the University of the Western Cape (UWC) online Library Database structure as the main database. Databases with a psychological and medical focus were searched including Academic Search Complete, APA PsycArticles, MEDLINE and SocIndex. An initial list of 15 keywords was identified from the available literature. These keywords were tested and combined into 12 Boolean phrases that were used within the first stage. The Smith-Fransciscus-Swartbooi (SFS) Quality Appraisal Tool developed by Smith et al.^23^ was utilised to appraise the identified articles in the eligibility phase.

Two reviewers were independently involved at each level of review to endorse and uphold operational thoroughness. In the appraisal stage, the reviewers’ scores were compared; any discrepancies in scores were identified and then discussed until agreement was reached. Initially, there were minor differences between the reviewers’ appraisals. Most of the differences were because of variances in scoring for the methodological rigor subsection of the SFS. The reviewers re-read the sub-sections of articles related to methodological rigour, observed the reason for the differences and discussed these until consensus was reached. Finally, once the differences were alleviated, articles with a score of 61% and above, the set threshold score in the ‘strong category’ on the SFS, were accepted to advance to the fourth phase, summation, where thematic meta-synthesis was used to systematically code and identify key themes.^24^

### Ethical considerations

The Humanities and Social Sciences Research Ethics Committee (HSSREC) at the University of the Western Cape provided approval to conduct the review HS20/5/6. The primary researcher was a registered master’s student, therefore able to access the online university library and resources for data collection. The review used published articles in the public domain; therefore, no further consent for access was required. Ethical considerations included adherence to the protocols ensuring accurate and thorough research procedures following strict, unbiased protocols, as specified above. Permission to utilise the appraisal tool was obtained from the main developer.

## Review findings

### Process results

#### Identification

The title search identified 1840 articles. After 1113 duplicates were removed, 727 articles were screened by title. Following this, 543 titles were omitted because they did not fulfil the inclusion criteria.

#### Screening

A total of 184 articles were screened by abstract, and 125 articles were excluded, mainly because of not having a focus on adult ADHD intervention approaches. Seven articles did not allow open access on the UWC website database and were therefore excluded.

#### Appraisal

In addition, 8 of the 52 articles were excluded after extraction before appraisal. Three were retrospective studies, four were not focused on intervention, and one article’s target population was children. Thus, only 44 articles were appraised.

The SFS appraisal tool consists of 42 questions divided into eight sections including the purpose of the intervention, intervention used, sample composition, ethics employed, instruments used, data analysis, results and conclusion. Only articles that fell in the 61% – 80% (strong category), as specified by the SFS, were retained for summation. Four articles were excluded because they did not achieve the 61% threshold score.

### Methodological standard of the studies

There were noticeable differences in the way that authors chose to contextualise and operationalise their studies as evident by their methodological choices, reporting of the results and discussions of the outcomes of the intervention approaches. It was noteworthy that none of the authors reported on or described their theoretical orientation as an operational frame for the intervention employed.

In contrast to this, most studies reported on the development of the intervention, including relevant background on the establishment of the specific intervention and provided motivation why it was thought to be effective. Most studies also mentioned previous studies where a similar intervention approach was utilised. The description of the intervention development might be considered as a way the authors contextualised the study.

Eighty-five per cent (*n* = 34) of the studies described the strategies employed in the intervention and reported on the implementation of the strategies. In studies that used a pharmacological approach, a description of the category of drug, amount used, dosage and period of the intervention was included. In studies with a non-pharmacological approach to intervention, an overview identified and discussed the specific goals of each session in the treatment programme, and the number of sessions was indicated.

Seventy-five per cent (*n* = 30) of the studies used probability sampling, most likely because of the high number of randomised control trials (RCT’s) that were performed. Participants were randomly assigned to groups in the majority of RCT studies. Contrary to an emphasis in literature that a parameter needs to be employed to determine sample size,^25^ it was noteworthy that 85% (*n* = 34) of the studies did not identify or report on the parameter used to determine sample size. In terms of the identification and use of measures to collect data, all studies gave a thorough description of the choice of instrument and the reason for this choice. Thirty-eight per cent (*n* = 15) studies employed the Connor’s Adult ADHD Rating Scale (CAARS) for pre- and post-testing.

All studies (*n* = 40) acknowledged the method of analysis and reason for its use. Thirty-five studies (*n* = 88%) did not report on the fidelity of the intervention implemented; thus, the degree to which the interventions followed an established protocol was mostly unidentified. This is an important information regarding the replicability of studies, speaking to adherence to methodological rigour. All studies had a clear conclusion section and identified and discussed limitations and made relevant recommendations for future focus in research studies. Ninety-five per cent (*n* = 38) of the studies received approval from an identifiable committee, obtained ethics clearance and mentioned that they obtained informed consent from participants that partook in the study.

### Types of intervention approaches to treat adult attention-deficit/hyperactivity disorder

Although this article’s main focus is to report on intervention approaches to treat adult ADHD, it needs to be acknowledged that adult ADHD often overlaps with other comorbid conditions such as mood disorders and anxiety disorders, intermittent explosive disorders, substance use disorders, antisocial personality disorders and other personality disorders.^1,4^ Thus, an integrative approach is needed to also attend to diagnosed co-morbidities such as anxiety and depression if they present in combination with adult ADHD.^1^

Identified intervention approaches to treat adult ADHD were divided into three categories: pharmacological intervention studies consisting of stimulants (*n* = 12) and non-stimulants (*n* = 8), and non-pharmacological intervention studies consisting of psychosocial (*n* = 13) interventions and interventions with an emphasis on neuro-stimulation techniques (*n* = 4).

#### Pharmacological interventions

The pharmacological studies included the use of two classes of stimulants, methylphenidate (MPH) and amphetamines such as lisdexamphetamine (LDX) and dextroamphetamine, targeting the central nervous system. Some of the identified adverse effects of stimulants use that were mentioned included sleeplessness, irritability, loss of appetite, decreased weight, gastrointestinal symptoms, headaches, heart tremors and anxiety. Further challenges identified were a non-reduction in symptomology and/or the risk of dependence or misuse of the stimulants. In situations where stimulants were contraindications, the use of non-stimulants was suggested. Non-stimulants used in the interventions were ATX, memantine and donepezil (cholinesterase inhibitors), as well as nicotinic agonists, which included ispronicline and pozanicline. Similarly, to the adverse effects mentioned above, it was suggested that although non-stimulants are not usually considered to be addictive in nature, adverse effects such as dry mouth, sleeplessness, nausea, loss of appetite, constipation and diminished libido were present. Notwithstanding these adverse effects, the non-stimulants were still considered as an effective choice of treatment for adult ADHD.^26,27^

Although pharmacological approaches were identified as the most used treatment modality for adult ADHD, the use of non-pharmacological approaches was also emphasised as an effective and/or alternative treatment option, mostly as an adjunct to pharmacological treatment.

#### Non-pharmacological approaches – Psychosocial interventions

Non-pharmacological approaches included brain mapping therapy (MAP) (*n* = 2), MAP and psychoeducation (*n* = 2), mindfulness-based training/DBT (*n* = 1), DBT (*n* = 2), CBT (*n* = 1), mindfulness-based cognitive therapy (MBCT) (*n* = 2), metacognitive therapy (MCT) (*n* = 1), client-centred therapy (CCT) (*n* = 1) and coping skills training/CCT (*n* = 1). The psychosocial interventions were all used either as the preferred stand-alone modality or in combination with other psychosocial interventions or with pharmacological treatment. A second category of non-pharmacological treatment was identified as treatments with an emphasis on neuro-stimulation. This entailed the stimulation of specific brain areas that showed abnormal activity in adults with ADHD.

#### Interventions with a focus on neuro-stimulation

Neuro-stimulation techniques included the use of a variety of techniques including neurofeedback and heart rate variability biofeedback (*n* = 1), bright light therapy (*n* = 1), transcranial direct current stimulation (*n* = 1) and quantitative electro-encephalography (qEEG)-informed neurofeedback (*n* = 1). The studies mostly utilised one of the stated approaches to the treatment of ADHD.

### Effectiveness of the treatment approaches

A single, combined, or multimodal approach to treatment was found in most of the intervention approaches identified in this review. Table 1^28^ presents a synopsis of the outcomes of the pharmacological intervention approaches.

**TABLE 1:** Outcome of pharmacological interventions.

Author/year	Modality and/or treatment	Outcomes in adults with ADHD
Agay et al., 2010	Methylphenidate	Improved aspects of cognitive performance and improvement in short-term memory
Arvidsson et al., 2019		Improved processing periods
Low et al., 2018		Better quality visual processing speed (alertness)
Nielson et al., 2017		Improved perceptual, cognitive and processing efficiency
Schrantee et al., 2016		Decreased cerebral blood flow in cortical areas; no mention of positive effects on ADHD symptoms
Verster et al., 2010		Improved declarative memory
Volkow et al., 2012		Reduction in symptoms of inattention, significant reduction in ADHD symptoms
Biederman et al., 2012	Osmotic-release methylphenidate	Significant decrease in ADHD symptoms
Chronis-Tuscano et al., 2010		Significant improvement in ADHD symptoms
Coppola et al., 2018	Atomoxetine	Significant reduction in the levels of impulsivity
Fan et al., 2017		Improved inhibitory control, heightened visual processing and improvement in clinical symptoms
Lin et al., 2015		Improved in impulsivity, hyperactivity and inattention
Ramos-Quiroga, 2014		Significant improvement in ADHD clinical symptoms
Nie et al., 2013	Methylphenidate and/or Atomoxetine (Comparison)	MPH and ATX were effective in enhancing executive functioning. ATX is more effective in improving spatial planning compared with MPH.
Nie et al., 2016		MPH and ATX improved intra-individual variability in reaction time and increased inhibitory control.
Adler et al., 2017	Lisdexamphetamine	Core ADHD symptoms improved significantly.
Brams et al., 2012		Core symptom reduction in majority of participants.
Franzen et al., 2013	Dextroamphetamine	Reduction of attention deficits and symptoms
Biederman et al., 2017	Memantine	Improvement in selective areas of executive functioning
Potter et al., 2014	AZD3480 (Ispronicline)	Significant improvements in inattention, impulsivity and hyperactivity
Jucaite et al.,2014	AZD1446 (Donepezil)	Well tolerated, but no significant improvement in core symptoms; significant increase in executive functioning
Apostol et al., 2012	ABT-089 (Pozanicline)	Effective and generally well-tolerated; improved core symptoms of ADHD.

*Source*: Extracted from: Wakelin C. A systematic review of recent interventions for adults with ADHD. Masters dissertation [homepage on the Internet]. University of the Western Cape; 2022 [cited n.d.]. Available from: http://hdl.handle.net/11394/9217

Note: All of the references are included in the thesis of Ms Wakelin and can be sourced from the thesis.

ADHD, Attention-deficit/hyperactivity disorder; MPH, methylphenidate; ATX, atomoxcitine.

Ten of the studies used a single medication to treat ADHD and reported improvement in impulsivity, inattention and hyperactivity, the core symptoms of adult ADHD. The five medications of choice employed in the studies included MPH (*n* = 5), dextroamphetamine (*n* = 1), ATX (*n* = 1), donepezil (*n* = 1) and memantine (*n* = 1). Three studies used ATX as the medicine of choice, three used MPH, two used LDX, while the last two used nicotinic agonists. Nine of the 22 studies failed to show improved core symptomatology but still reported on other improvements, including better visual processing speed, reduced impulsiveness, increase in short-term memory, attentiveness and a marked improvement in executive functioning. Individuals reported increased functioning in home and work contexts.

The given results emphasised that a variety of medications proved effective in dealing with the core symptoms of ADHD in adults and that the decision of which medication to use is determined by idiosyncratic features of the individuals who partook in the studies. Lastly, one study^29^ used MPH as the choice of intervention to identify and report on the differential effects of MPH use in adults and children. Although the focus was supposed to be on the effectiveness of the medication to reduce ADHD symptoms in adults and children, reported results focused on MPH’s ability to increase cerebral blood flow in cortical areas. Other studies^30,31^ explained that the use of MPH and the effect that it has on the brain can lead to adverse symptoms such as headaches, seizures, encephalopathy and stroke.

#### Comparison of pharmaceutical drugs

The remaining two studies (of the 22 that had a pharmacological approach to symptom reduction) used a comparative approach, comparing one medication of choice with another to determine effectiveness.^26,27^ Both studies compared MPH with ATX to ascertain which would be more effective in symptom reduction in adult ADHD populations. According to the first study,^26^ the results indicated improved executive functioning from both MPH and ATX; however, participants taking ATX showed more significant improvement in spatial planning. The second study^27^ reported a similar improvement in reaction time and inhibitory control for both pharmaceuticals. It seems as if both ATX and MPH are seen as popular choices in the treatment of adult ADHD and are recommended as frontline treatment modalities.

Table 2^28^ provides a synopsis of the outcomes of non-pharmacological interventions with a psychosocial focus. A decrease in inattention, impulsivity and hyperactivity (core symptoms of ADHD) were found in seven of the nine studies that used a single therapeutic approach as treatment modality. The modalities used were DBT (*n* = 2), mindfulness and variations thereof (*n* = 2), and CBT and variations (*n* = 2). Divergently, two therapeutic approach studies^32,33^ failed to report improvement in inattention, hyperactivity and impulsivity. The single study^32^ utilising MAP did not show a decrease in the core symptoms categories but still showed a reduction in inattention, resulting in reported enhancement of the individuals’ quality of life.^32^ Additionally, the study^32^ using CBT as an intervention also failed to report improvement in the core symptoms categories, yet still indicated better attention, sleep and a reduction in symptoms of depression. Thus, utilising MAP and CBT simultaneously was found to be useful. It needs to be noticed that adults with ADHD do not always present with impulsivity, inattention and hyperactivity. They may be diagnosed with the predominantly inattentive or predominantly hyperactive/impulsive subtypes and thus may not require an intervention that focusses on the reduction of all three stated components. An enquiry into the specific symptomology of the individual is thus essential before a decision is made about the most suitable intervention for the individual.^33^

**TABLE 2:** Outcomes of interventions with a psychosocial focus.

Modality and/or treatment	Author and year	Outcomes
Mindfulness meditation (MAP)	Mitchell et al., 2017	Improved ADHD and executive functioning symptoms, as well as reduction in emotion dysregulation.
	Bueno et al., 2015	Improved inattention, mood and quality of life for patients/control groups.
MAP/psychoeducation	Bachmann et al., 2018	Both generated a significant decrease in ADHD core symptoms and an improvement in task performance.
	Hoxhaj et al., 2018	Improved self-concept, quality of life, overall mental health, depression decreased and improved mindfulness.
Mindfulness-based training/DBT	Edel et al., 2017	MBT and DBT had similar reduction in ADHD core symptoms
DBT	Fleming et al., 2015	Improvement in mindfulness and self-efficacy, executive functioning and quality of life
	Morgensterns et al., 2016	Enhanced well-being, ability to be mindful, better emotional regulation and quality of life.
CBT	Solanto et al., 2018	Improvement in inattentive symptoms, sleep and depressive symptoms
MBCT	Gu et al., 2018	Improved ADHD core symptoms and decrease in anxiety and depression, assisted with higher levels of mindfulness and neuropsychological performance
	Hepark et al., 2015	Significant reduction in ADHD symptoms, improvement in executive functioning, mindfulness
Meta-cognitive therapy	Solanto et al., 2010	Improved overall core symptoms of ADHD
CCT	Stern et al., 2014	Marked improvements in executive functioning and mindfulness competencies
Coping skills and/or CCT	Bettis et al., 2017	Improved executive functions, reduced ADHD symptomatology and improved occupational performance, decrease in social stress, executive function difficulties and anxiety symptoms.
		CCT programme reported improved ADHD symptoms in comparison with coping skills.

*Source*: Extracted from: Wakelin C. A systematic review of recent interventions for adults with ADHD. Masters dissertation [homepage on the Internet]. University of the Western Cape; 2022 [cited n.d.]. Available from: http://hdl.handle.net/11394/9217

Note: All of the references are included in the thesis of Ms Wakelin and can be sourced from the thesis.

ADHD, Attention-deficit/hyperactivity disorder; MBT, mindfulness-focused training; DBT, dialectical behaviour therapy; CBT, cognitive-behavioural therapy; CCT, client-centred therapy; MBCT, mindfulness-based cognitive therapy.

Six studies described other benefits that individuals experienced after psychosocial interventions, namely an improvement in their executive functioning^34,35,36,37^ and the ability to be aware and mindful.^36,38,39^ An improvement in quality of life, mental well-being, mood and a reduction in depressive symptoms were also outcomes of the interventions in these studies. This might link to psychosocial approaches assisting patients to self-regulate, which pharmacotherapies fail to address.^35,37^

#### Comparison of therapeutic approaches

Four studies used a comparative stance in that one type of intervention was compared with another, to establish which intervention had the most positive outcome for adults with ADHD. Two of these studies compared MAP with psychoeducation to reduce ADHD symptomology.^40,41^ No significant difference was found in the use of these treatment modalities; results indicated that both were effective in reducing the core symptoms of ADHD. This is an interesting result as these two modalities are vastly different in their approaches to intervention, which seems to indicate that a wide variety of techniques can assist with ADHD symptom reduction. Additionally, improved task performance, increase in self-concept, quality of life, overall mental status and mindfulness were also consistent across both studies.

One study^42^ compared MBT and DBT as treatment modalities and found that both were effective in reducing core symptoms of ADHD. Finally, one study compared CCT and coping skills in management of ADHD in adults.^43^ Although both decreased social stress significantly and enhanced executive functioning and reduced anxiety in participants, CCT was found to be superior to enhance coping skills to deal with some of the symptoms of ADHD. It is possible that coping skills as an intervention strategy may be too simplistic in its ability to reduce ADHD symptoms, thereby losing sight of the complex nature of this disorder.

Table 3^28^ provides a synopsis of the outcomes of interventions with a focus on neuro-stimulation. The outcome indicated improvement in core symptoms in three of the four studies.^44,45,46^ The remaining study reported improved inhibition of impulsivity.^47^ These insights remain under-investigated as only four studies were identified and reported upon neuro-stimulation as an intervention. The limited number of articles found might be because of the previously specified search criteria used in this review.

**TABLE 3:** Outcomes of interventions with a focus on neuro-stimulation.

Modality and/or treatment	Author and/or year	Outcomes
Z-score neurofeedback and heart rate variability biofeedback exercises	Groeneveld et al., 2019	Improved symptoms of ADHD and task performance
Bright light therapy	Fargason et al., 2017	Decrease in core symptoms of ADHD
Transcranial direct current brain stimulation	Allenby et al., 2018	Reduced impulsivity
QEEG-informed neurofeedback	Arns et al., 2012	Significant decrease in core symptoms and comorbid depressive symptoms

*Source:* Extracted from: Wakelin C. A systematic review of recent interventions for adults with ADHD. Masters dissertation [homepage on the Internet]. University of the Western Cape; 2022 [cited n.d.]. Available from: http://hdl.handle.net/11394/9217

Note: All of the references are included in the thesis of Ms Wakelin and can be sourced from the thesis.

ADHD, attention-deficit/hyperactivity disorder; QEEG, quantitative electroencephalography.

## Implications and recommendations

The intervention methods described in this review were found to be effective in treating the core symptoms of adult ADHD. Some of the outcomes focused on the successful reduction of impulsitivity, inattention and hyperactivity, while others were effective in reducing either one or two core symptoms and other symptoms linked to comorbidities such as depression. Methylphenidate and ATX were identified as the first-line medication to use for adult ADHD. As a result of the potential for mismanagement, contraindications, adverse effects and possibility of ineffectiveness, non-pharmacological interventions were identified as an alternate treatment modality or as adjunct to pharmacology. The treatment options that were deemed most effective were DBT, CBT and variations on these, as well as interventions that were mainly focused on mindfulness techniques.

Interventions that focused purely on neuro-stimulation were also recommended; however, very few studies were found using this treatment approach. It was interesting to note that both pharmacological and non-pharmacological intervention approaches improved individuals functioning in the executive domain, reduced challenges experienced with sleep and relieved symptoms associated with depression and anxiety. These methods also enhanced memory functioning, led to an increase in emotional regulation and overall improved individuals’ quality of life. Overall treatments proved useful in terms of the management of comorbidities known to be present in many adults with ADHD.

This review had specific limitations. Language bias impacted the amount of data available for analysis, as only English studies were considered. Additionally, non-English studies with significant results were more likely to be reported in English-language journals rather than those with non-significant results; thus, suggesting possibility of publication bias. Publication bias also restricted the sampling frame, as the review used peer-reviewed journal articles only. However, studies are not always published in peer-reviewed journals but may be presented as theses, book chapters, conference abstracts or might remain unpublished.

## Conclusion

The primary contribution of the study is that it combined available literature on the most recent interventions and its outcomes available for adults with ADHD, adding to the existing information on this topic. The literature further emphasised the need for well-researched interventions that reduce the core symptoms and associated features of Adult ADHD. These interventions can also facilitate changes to general policy documents on mental well-being, inclusive of workplace strategies that deal with challenges that might present itself in the workplace environment.
